# Multiple food-borne trematodiases with profound systemic involvement: a case report and literature review

**DOI:** 10.1186/s12879-019-4140-y

**Published:** 2019-06-14

**Authors:** Lun Li, Xinchao Liu, Baotong Zhou, Shimin Zhang, Geng Wang, Guotao Ma, Ruxuan Chen, Yang Zou, Wei Cao, Taisheng Li

**Affiliations:** 1 Department of Internal Medicine, Peking Union Medical College Hospital, Chinese Academy of Medical Sciences, Beijing, 100730 China; 2 Department of Infectious Diseases, Peking Union Medical College Hospital, Chinese Academy of Medical Sciences, #1 Shuaifuyuan, Dongcheng District, Beijing, 100730 China; 3 Department of Clinical Laboratory, Peking Union Medical College Hospital, Chinese Academy of Medical Sciences, Beijing, 100730 China; 4 Department of Cardiac Surgery, Peking Union Medical College Hospital, Chinese Academy of Medical Sciences, Beijing, 100730 China; 5 Beijing Institute of Tropical Medicine, Beijing Friendship Hospital, Capital Medical University, Beijing Key Laboratory for Research on Prevention and Treatment of Tropical Diseases, Beijing, 100050 China

**Keywords:** Trematodiases, Food-borne, Ectopic lesion

## Abstract

**Background:**

Food-borne trematodiases are an important group of neglected global diseases. Affected patients in regions with low prevalence usually experience delayed diagnosis, especially when presenting with atypical clinical symptoms. Here, we presented a rare case of a Chinese patient infected with three food-borne trematodiases.

**Case presentation:**

A 42-year-old man presented with diarrhea, lower extremity edema, and symptoms of cardiac dysfunction. He had a history of intermittent consumption of raw freshwater fishes for 6–7 years. Upon evaluation, he had eosinophilia, anemia, intrahepatic bile duct dilatation and a growing space-occupying lesion in the left atrium. The patient underwent a cardiac surgery which revealed an endocardial hematoma due to mechanical injuries. Imaging investigations also revealed intracranial and pulmonary lesions. A total of three trematodiases were diagnosed based upon microscopic stool examination, from which eggs of *Clonorchis sinensis*, Heterophyidae and Echinostomatidae were identified. Deposition of *Clonorchis sinensis* eggs was also observed from ileocecal squash slides. The patient was successfully treated with three cycles of praziquantel.

**Conclusions:**

Food-borne trematodiases may present with systemic involvement. Patients with dietary history of high risk or atypical ingestions should be evaluated for parasitic infection, even in non-endemic areas.

**Electronic supplementary material:**

The online version of this article (10.1186/s12879-019-4140-y) contains supplementary material, which is available to authorized users.

## Background

Foodborne parasitic diseases remain largely underestimated worldwide, with the highest prevalence reported in East and Southeast Asia [1]. Food-borne trematodes are classified into liver, intestinal, and lung flukes, based on their primary sites of infection. However, a few flukes may migrate substantially within the body, leading to systematic damage involving the skin, abdomen, heart, or central nervous system (CNS) [1–3]. Uncommon clinical manifestations may potentially delay diagnosis and treatment in non-endemic areas. Although immunodiagnostic techniques and molecular methods can present important clues, microscopic examination is always the definitive means of diagnosis. Here we report a case of a critically ill patient infected with multiple trematodes, complicated by ectopic parasitism.

## Case presentation

A 42-year-old man presented to the emergency room with intermittent diarrhea for over four months and progressive lower extremity edema for three months. He had 5–6 bowel movements per day, with no obvious fever or abdominal discomfort. Progressive pitting edema of the bilateral legs was noticed one month later, accompanied by a decline in exertional tolerance. The patient lost approximately 15 kg over this period of time. A series of echocardiographic examinations had revealed a growing mass in his left atrium of uncertain origin, which grew from 25 × 22 to 60 × 54 mm within 3 months. His previous medical history was remarkable for poorly controlled type 2 diabetes mellitus complicated by diabetic nephropathy, retinopathy and peripheral neuropathy. He also had a five-year history of major depressive disorder without regular treatment. He lived with his mother and sister in the city of Qingdao, Shandong Province, and denied any recent travel history. The patient’s sister reported that he had intermittently consumed raw river fish for as long as 6–7 years before onset of this episode. Due to the mental status of the patient, his medical history was also obtained and confirmed by his mother and sister who lived with him.

On admission, the patient appeared emaciated and anemic. He was afebrile, his blood pressure was 93/67 mmHg, and heart rate 102 bpm. He was moody, disoriented, and slightly hypoxemic with an oxygen saturation of 90% at room air. A grade II diastolic rumbling murmur was heard at the apex. Examination of his lungs and abdomen was otherwise unremarkable. Decreased myodynamia of the bilateral limbs was appreciated, more significant on the left side, with a positive left Babinski sign.

**Fig. 1:**
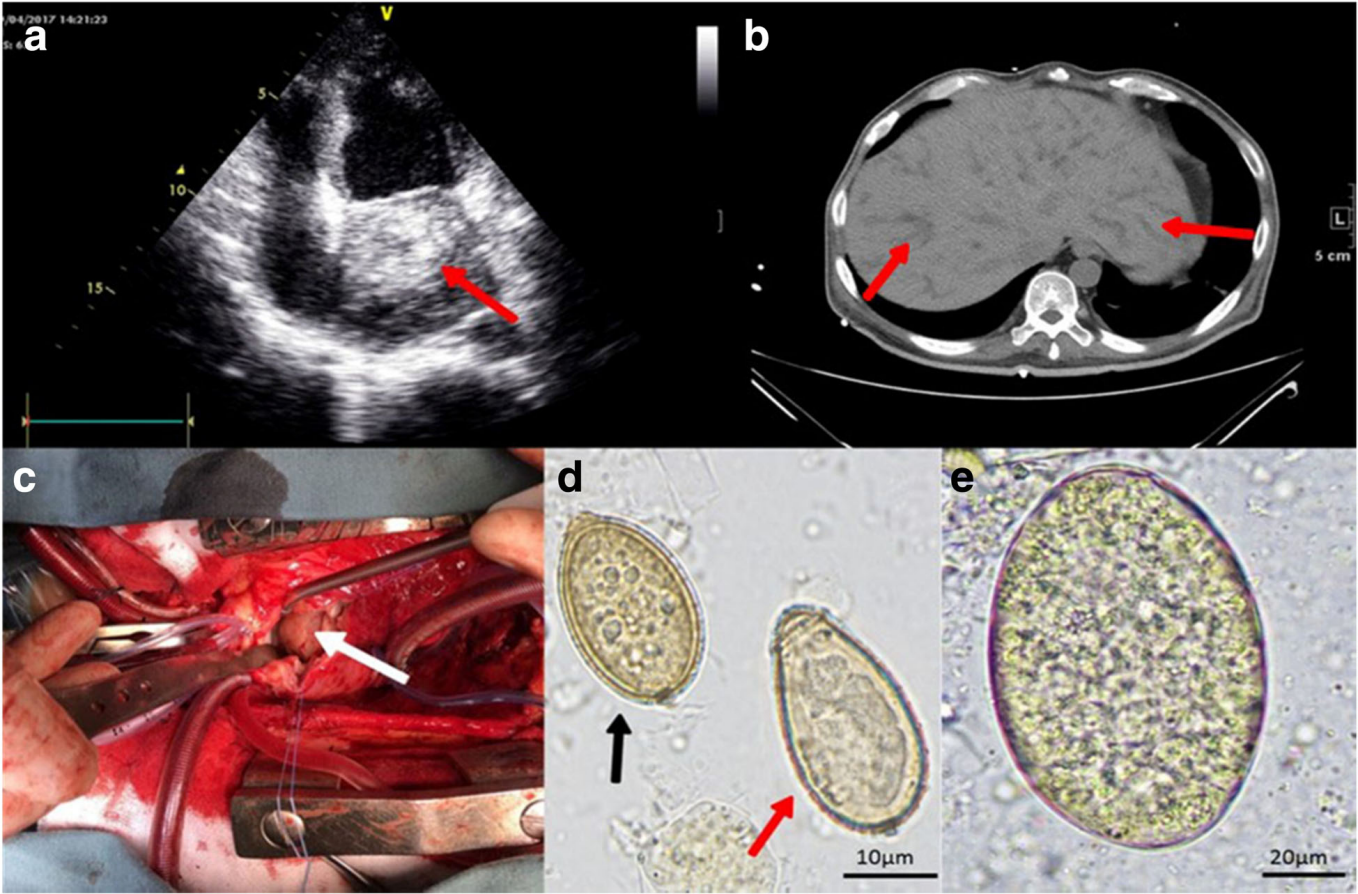
Imaging, surgical and pathogenic findings in the patient. (**a**) A space-occupying lesion (red arrow) was observed in the left atrium measured 60 × 54 mm by cardiac ultrasonography. (**b**) Abdominal CT scan showed extensive intrahepatic bile duct dilatation (red arrow). (**c**) The round cardiac mass (white arrow) in the left atrium was exposed in the surgery, which was an endocardial hematoma close to the posterior mitral valve. (**d**) The egg of *C. sinensis* (red arrow) was firstly identified in the stool samples, and later a smaller egg (black arrow) consistent with that of heterophyid trematodes was distinguished from *C. sinensis*. (**e**) Larger-sized eggs in the stool samples were consistent with those of the Echinostomatidae family

Initial laboratory assessment revealed peripheral eosinophilia (eosinophils 6.64 × 10^9^/L) and anemia (hemoglobin 85 g/L). The level of gamma-glutamyl transpeptidase was elevated at 1093 U/L, and alkaline phosphatase was 666 U/L, with no apparent hyperbilirubinemia (Table 1). Serial of electrocardiograms showed paroxysmal atrial fibrillation. A bedside echocardiogram was immediately arranged, and a space-occupying lesion measuring 60 × 54 mm was identified in the left atrium with a slight pericardial effusion (Fig. 1a). The left ventricular ejection fraction (LVEF) was moderately reduced at the level of 55%. The initial chest CT showed scattered bilateral pulmonary infiltrations and pleural effusion, with a cavity in the right upper lobe. Abdominal CT scan showed intrahepatic bile duct dilatation with no obvious obstruction (Fig. 1b). A CT scan of the head was also preformed due to recurrent episodes of seizures during hospitalization, and showed low density lesions in bilateral corona radiate.

**Table 1:** Laboratory data for the case patient

Test	Case patient	Reference Range, Adults
Leukocytes, ×10^9^/L	14.57	3.5–9.5
Eosnophils, ×10^9^/L	6.65	0.02–0.50
Eosnophils, %	45.6	0.5–5
Erythrocyte, × 10^12^/L	3.1	4–5.5
Hemoglobin, g/L	85	120–160
Platelets, ×10^9^/L	325	100–350
Albumin, g/L	37	35–52
Total bilirubin, μmol/L	5.6	5.1–22.2
Aspartate aminotransferase, IU/L	22	15–40
Alanine aminotransferase, IU/L	12	9–50
Gamma-glutamyl transpeptidase, IU/L	1473	10–60
Alkaline phosphatase, IU/L	905	45–125
Urea, mmol/L	9.65	2.78–7.14
Creatinine, μmol/L	97	59–104

The initial differential diagnosis included infectious endocarditis, Löffler endocarditis, hypereosinophilic syndrome, parasitic infection and hypersensitive reactions to recent medication. Although no evidence of fungus was identified from qualified sputum samples, sulperazon and caspofungin were initiated empirically based on the CT manifestation and history of uncontrolled diabetes. Supportive care was also initiated. A multi-disciplinary discussion was held, and consensus was achieved regarding the urgency of cardiac surgery, both to alleviate progressive heart failure and to obtain a tissue specimen for diagnostic purposes. The patient underwent the surgery two weeks following admission. Surprisingly, the cardiac mass turned out to be an endocardial hematoma, possibly due to mechanical perforation of the left ventricular posterior wall close to the base of posterior mitral valve (Fig. 1c). The valves were otherwise intact. Pathological review of the specimen revealed nonspecific inflammation and all pathogenic examinations turned out to be unremarkable.

The surgical findings largely ruled out endocarditis of infectious or autoimmune origin, nor did they reveal any evidence of eosinophilic infiltration. Given the systemic involvement and the dietary habits mentioned by his family, we began to consider the possibility of parasitic infection with systemic involvement. A more detailed history confirmed raw or half-raw consumption of freshwater fish and shrimps, frogs, tadpoles and snake gall bladders nearly every month in recent years as a result of his altered mental status. Further tests were warranted. Multiple stool samples were sent for microscopic examination by direct fecal smear, and various eggs were identified. Eggs of *Clonorchis sinensis* were first identified (measuring 27-30 μm × 16-18 μm, Fig. 1d), at a count of 3–5/low-power (LP) field. Clonorchiasis was therefore confirmed, which partially explained the patient’s chronic diarrhea and dilatation of intrahepatic bile ducts. However, this liver fluke rarely causes such widespread organ damage. A few days later, another type of egg smaller than that of *C. sinensis* was identified, which was morphologically most consistent with that of heterophyid trematodes (measuring 23-26 μm × 13-15 μm, Fig. 1d). The eggs of Heterophyidae were much fewer in number, and required 10–20 LP field to find one. Moreover, several large-sized eggs were also observed (measuring 100-110 μm × 60-70 μm), consistent with those of the Echinostomatidae family (Fig. 1e). Approximately five eggs of the Echinostomatidae could be identified per fecal smear. Further evaluations included a contrast-enhanced cerebral magnetic resonance imaging test with angiography, which revealed multiple long T2 densities in the centrum semiovale with no obvious vascular involvement. Lumbar puncture was performed and showed moderately elevated intracranial pressure at 210 mmH_2_O and a slightly elevated protein level in the cerebral spinal fluid. A colonoscopy was apparently normal, but deposition of *C. sinensis* eggs was later observed on ileocecal squash slides. Specific PCR band patterns of *C. sinensis* were later observed in samples of the ileocecal tissues and stool, but not in the sputum or heart tissues. DNA of heterophyid trematodes was not detected from any obtained samples. The primers of PCR were designed according to previous studies (Additional file 1: Table S1).

Once the diagnosis of triple trematodiases was established, treatment with praziquantel was started. Due to the comprehensive involvement and the concern for hypersensitivity reaction, a reduced oral dose of praziquantel at 25 mg/kg/d was initiated for the first 10 days combined with low-dose dexamethasone. The patient tolerated this regimen well and received the full dose of 75 mg/kg/d for ten days each month for another consecutive two months. His diarrhea gradually resolved with a steady improvement in nutritional status and cardiac function. Continuous surveillance of his stool samples revealed no further trematode eggs since the second month of treatment. Follow-up CT scans showed remission of pulmonary and liver lesions, while enhanced MRI showed absorption of previous abnormal signals in centrum semiovale.

## Discussion and conclusions

Clonorchiasis is a widespread foodborne infectious disease and has been reported in 24 provinces in China. However, the prevalence varies, and infections of families Echinostomatidae and Heterophyidae are rarely reported especially in the northern areas [4]. Infections are most frequently acquired through eating uncooked freshwater fish or other aquatic products containing encysted organisms. The present patient represented a very rare case of multiple trematode co-infections including *C. sinensis*, Heterophyidae and Echinostomatidae, confirmed by parasitological stool examination. His clinical manifestations provided an indication of the sites, load and intensity of parasitic infection, as well as the host immune status.

Mild to moderate infection of any of the three trematodes may present with unspecific symptoms, while severe forms may lead to symptoms such as fatigue, weight loss, anemia, abdominal pain and diarrhea [5, 6]. The triple trematodes infection, especially the heavy burden of *C. sinensis* in this case, is sufficient to explain the gastrointestinal manifestations, emaciation and anemia of this patient. Characteristic cholestasis with intrahepatic bile duct dilatation caused by clonorchiasis was also observed in this patient. However, involvement of the heart, brain and lungs are not commonly seen in trematodes infection, yet we did not find any evidence of autoimmune diseases, malignancies or other infections which could explain such comprehensive damage. As far as we know, among the three kinds of trematodes we identified, Heterophyidae is the only one whose eggs can enter the blood circulation and cause ectopic parasitism, especially in immunocompromised hosts. Until now, ectopic infection has not been described in clonorchiasis or echinostomiasis. Based on this knowledge and the patient’s response to treatment, we deduced that it was most probable that heterophyid ectopic parasitism led to lesions of the brain, lung and heart in this case (Table 2). The most frequently reported sites of infection for heterophyidiasis are the heart valves and the brain, which could be potentially fatal [1–3, 7–9]. However, it was reasonable to hypothesize that attachment of heterophyid eggs to the surface of the endocardium could cause necrosis and subsequent perforation, resulting in the endocardial hematoma.

**Table 2:** Trematodes infection and related manifestation for the case patient

Parasitic sites	Symptom/Manifestation	Trematode species	Diagnostic evidence	Possibility
Biliary system	Intrahepatic bile duct dilatation and liver damage	*C. sinensis*	Eggs identified and PCR positive in stool	Proven
Gastrointestinal tract	Diarrhea and weight loss	*C. sinensis*	Eggs identified and PCR positive in stool; Eggs deposited in ileocecal	Proven
		Heterophyidae	Eggs identified in stool	Proven
		Echinostomatidae	Eggs identified in stool	Proven
Heart	Endocardial hematoma and heart failure	Heterophyidae	Mechanic perforation of the ventricular wall	Probably
Brain	Seizures and decreased myodynamia	Heterophyidae		Possibly
Lung	Infiltrations and cavity	Heterophyidae		Possibly

The most straightforward and classic way for diagnosing parasitic infection is the detection of eggs or the parasite itself via morphological examination, as we did in this case. The detection of eggs in the stool is the standard for diagnosis of trematodiases. Detailed identification by means of delicate microscopic examination revealed *Metagonimus yokogawai* as the most likely species. However, further specification of Echinostomatidae was not possible through visual speculation. Immunodiagnostic techniques and molecular methods have been developed as an important supplement to diagnosis. However, false negative results are possible due to the testing methods, disease severity and pathogenesis of the infection. Moreover, cases with a low burden of eggs and/or unsuccessful cracking of eggs during DNA extraction can also lead to amplification failure on molecular testing, which might explain the difficulty achieving greater specificity in terms of each organism in this case [10, 11]. Nevertheless, ectopic infiltration and subsequent necrosis of surrounding tissue remained the most probable explanation for the mechanical injuries leading to this patient’s heart failure.

Praziquantel is the drug of choice for several species of trematodes including clonorchiasis from liver fluke infection, as well as heterophyiasis and echinostomiasis from intestinal fluke infection. The recommended dosage of 25 mg/kg three times daily for 2–3 days can be highly efficacious for clonorchiasis [1], but patients with a heavy pathogen load may require multiple courses [12]. Moreover, a single oral dose of 10-20 mg/kg praziquantel is reported to be sufficient for a treatment of heterophyiasis and echinostomiasis, but no prior reports have involved such diffuse and multiple fluke infection. Therefore, we adopted a progressive strategy with three cycles of therapy (starting with a low-dose praziquantel cycle combined with dexamethasone), to reduce allergic reaction and recurrence.

The patient described here represents a very rare case of multi-fluke infection with systemic involvement. Parasitic infection is one of the essential differential diagnoses of eosinophilia, but atypical manifestations may lead to delays or neglect in considering the diagnosis. Physicians in non-endemic areas need to raise their awareness of and keep in mind the signs of fluke infection. In particular, parasitic infection should be considered in cases with significant eosinophilia or relevant risk factors in the patient’s history. Immunodiagnostic techniques and molecular methods are newer alternatives for the detection of parasites in fecal samples, but microscopic examination remains the definitive diagnostic tool.

## Additional file

Additional file 1: **Table S1.** Primers for amplification by polymerase chain reaction (PCR). (DOCX 19 kb)

## Data Availability

Essential data and materials were presented in this manuscript. More clinical, laboratory, and imaging data are available from the corresponding author on reasonable request.

## Abbreviations

CNS: Central nervous system
CSF: Cerebral spinal fluid
LVEF: Left ventricular ejection fraction
PCR: Polymerase chain reaction
