# Supplementary material for: Multiple food-borne trematodiases with profound systemic involvement: a case report and literature review
Source: BMC Infect Dis. 2019 Jun 14;19:526. doi: 10.1186/s12879-019-4140-y (PMC6570942; doi:10.1186/s12879-019-4140-y)
Supplement: Supplementary file 1 — Table S1. Primers for amplification by polymerase chain reaction (PCR). (DOCX 19 kb) [file 12879_2019_4140_MOESM1_ESM.docx]

Supplemental Table 1 Primers for amplification by polymerase chain reaction (PCR)

| Species | Designation | Primer sequence (5’ to 3’) | Product size (bp) |
| --- | --- | --- | --- |
| *C. sinensis* [1] | CS ITS2 F | ACTATCACGAACGCCCAAA | 265 |
|  | CS ITS2 R | CTGAAGCCTCAACCAAAG |  |
| *M. yokogawai* [2] | my1 F | CATGTGCGGGTAGTCGTG | 648 |
|  | my2 R | TCATGTCGATCAACTGGGAAC |  |
| *H. taichui* [2] | ht1 F | ATATTGTTGCCTCCATGGCTGAC | 279 |
|  | ht6 R | CTGTACGTAGGTACACGTGTG |  |
| *H. taichui / H. pumilio* [3] | 3S F | GGTACCGGTGGATCACTCGGCTCGTG | 500 |
|  | A28 R | GGGATCCTGGTTAGTTTCTTTTCCTCCGC |  |
| *O. viverrini /C. sinensis /* | Heter ITS2 F | CTTGAACGCACATTGCGGCCATGGG | 380~530 |
| *H. pumilio / H. taichui* [4] | Heter ITS2 R | GCGGGTAATCACGTCTGAGCCGAGG |  |
| Heterophyidae [5] | Het 18S F | TCATATGCTTGTCTCAGA | ~1800 |
|  | Het 18S R | ACGGAAACCTTGTTACGA |  |

**References:**

1. Zhang Y, Tong R, Zheng Q, Xie M, Cao J. Detection of *Clonorchis sinensis* by PCR and Real-Time PCR. Parasitoses and Infectious Diseases. 2008; 6(1): 9-11.

2. Mei X, Li S, Kang Y, Shi Y, Huang T, Chen Z, Huang W. Establishment of Duplex PCR for Identifying Metagonimus yokogawai and Haplorchis taichui. Chin J parasitol Dis. 2015; 33(3): 176-180.

3. Mei X, Li S, Hu C, Huang T, Chen Z, Huang W. China Animal Husbandry and Veterinary Medicine. 2015; 42(8): 1943-9.

4. Sato M, Thaenkham U, Dekumyoy P, Waikagul J. Discrimination of O. viverrini, C. sinensis, H. pumilio and H. taichui using nuclear DNA-based PCR targeting ribosomal DNA ITS regions. Acta Tropica 2009; 109(1): 81-3.

5. Dzikowski R, Levy MG, Poore MF, Flowers JR, Paperna I. Use of rDNA polymorphism for identification of Heterophyidae infecting freshwater fishes. Dis Aquat Organ 2004; 59(1): 35-41.
